# Supplementary material for: A monomeric mycobacteriophage immunity repressor utilizes two domains to recognize an asymmetric DNA sequence
Source: Nat Commun. 2022 Jul 14;13:4105. doi: 10.1038/s41467-022-31678-6 (PMC9283540; doi:10.1038/s41467-022-31678-6)
Supplement: Supplementary file 1 — Supplementary Info File #1 [file 41467_2022_31678_MOESM1_ESM.pdf]

Supplementary Information for:

A Monomeric Mycobacteriophage Immunity Repressor Utilizes Two Domains to Recognize an Asymmetric DNA Sequence

Reliza J. McGinnis[1, ^], Chad A. Brambley[2], Brandon Stamey[1], William C. Green[1], Kimberly N. Gragg[1], Erin R. Cafferty[1, &], Thomas C. Terwilliger[3], Michal Hammel[4], Thomas J. Hollis[5], Justin M. Miller[2], Maria D. Gainey[1*], and Jamie R. Wallen[1*]

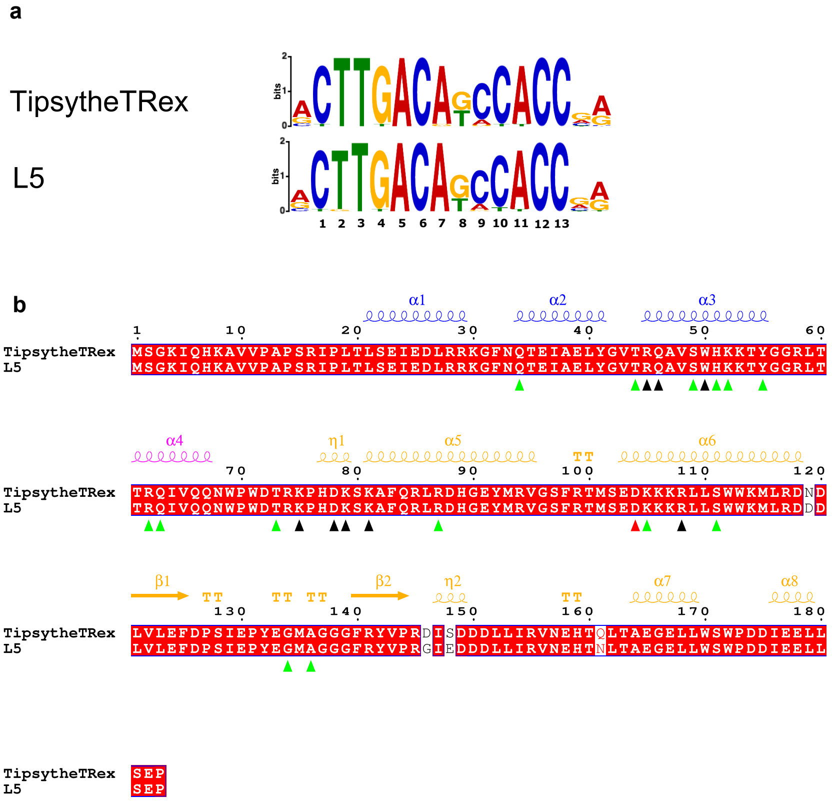

Supplementary Figure 1: **Comparison of L5 and TipsytheTRex sequences**. **a**) Shown is the consensus motif for the operator/stoperator sites identified in the genomes of L5 and TipsytheTRex using the MEME suite. Each nucleotide of the consensus sequence is numbered. **b**) Alignment of repressor sequences from TipsytheTRex and L5. The secondary structure is from the TipsytheTRex crystal structure and is both colored by domain and labeled as in Figure 1a. Black arrows indicate residues that contact DNA bases in the repressor structure, while green arrows designate residues that contact the DNA backbone. D104, which interacts with R108, is depicted with a red arrow. The TipsytheTRex and L5 protein sequences are 98% identical, with all DNA binding residues conserved. The figure for panel **b**) was generated using ESPript v 3.0[1].

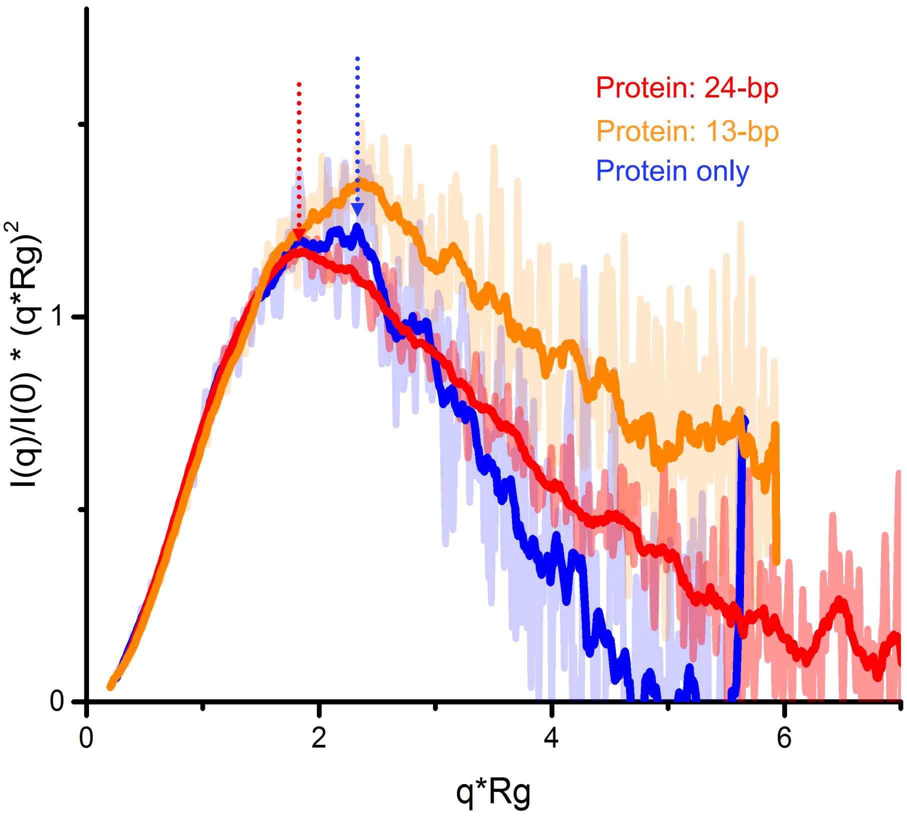

Supplementary Figure 2. Comparison of Kratky plots indicates increased rigidity of the repressor:DNA complex. Normalized Kratky plots for the repressor only (blue trace), repressor:13-bp DNA complex (orange), and repressor:24-bp DNA complex (red). The arrows highlight Kratky plot maxima that indicate increased rigidity of the protein upon DNA complexation. For better visualization, smoothing of the raw data were applied, where light colors represent the raw data, while the dark colors represent smooth curves.

a

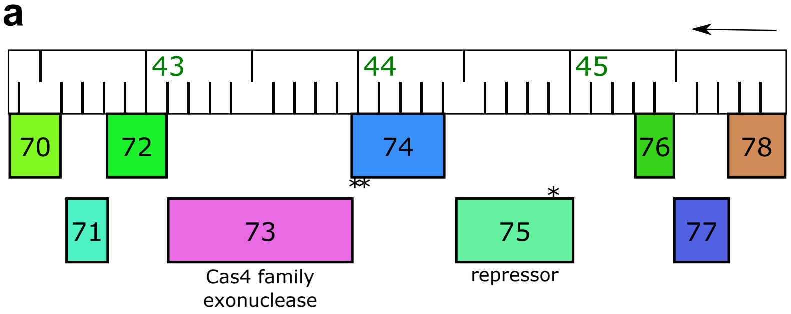

b

	Genome Position, Mutation	Gene	Amino Acid Mutation	EOP
REM1	43,988, A to G	74	M142T	$5.4x10^{-1}$
REM2	44,915, C to A	75	E36Stop	1.7
REM3	43,980, C to A	74	A145S	$9.5 x10^{-1}$
REM4	43,988, A to G	74	M142T	$8.4 x10^{-1}$
REM5	43,988, A to G	74	M142T	1.2

c

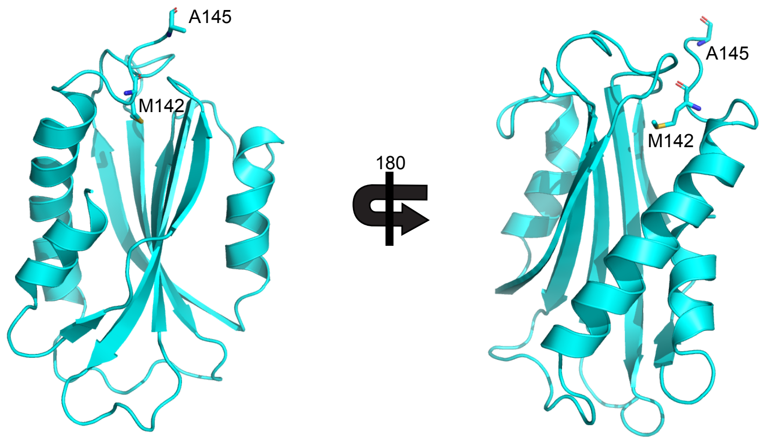

Supplementary Figure 3. Whole-genome sequencing of repressor escape mutants. The full genomes of wild-type TipsytheTRex and five repressor escape mutants (REMs) were sequenced and assembled. **a**) The location of the single point mutations that were found in each REM as compared to the wild-type stock are represented on the genome map by asterisks. The arrow indicates the direction of transcription. The ruler corresponds to the location in the genome in kb pairs, and genes are represented by colored boxes. Of the nine genes shown in the figure, only genes 73 and 75 have assigned functions. **b**) The location of the single point mutations found in each REM are shown along with the mutation conferred and the efficiency of plating (EOP). In addition to the mutations listed, the REM5 assembly was mixed A/G at position 23,361 of the genome. This results in a wild-type/N495S mixture for the gene 30 minor tail protein. **c**) AlphaFold model for TipsytheTRex gene product 74, with an average plDDT (confidence) of 75.3. The model is colored cyan and shown in cartoon, with the two residues (M142 and A145) mutated in the REMs shown as sticks. These two residues lie in a loop region at the very C-terminal end of the protein. Source data are provided in the Source Data file.

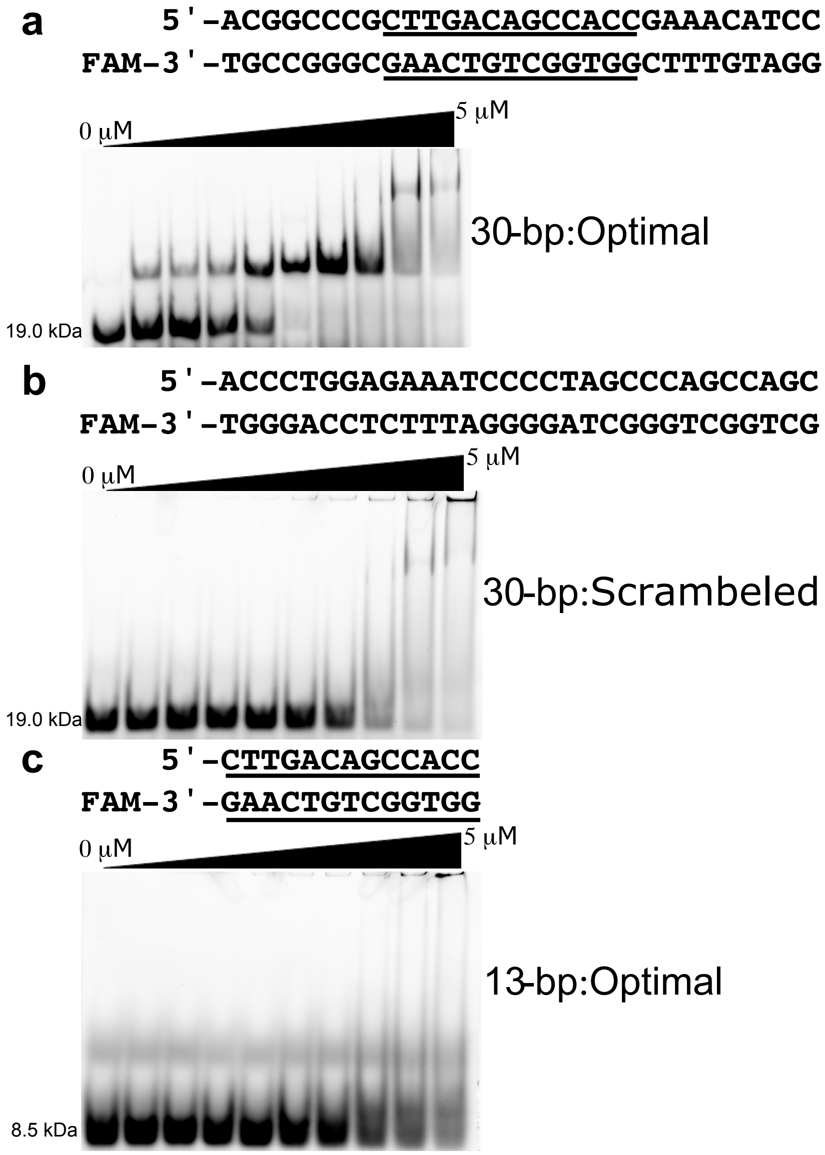

Supplementary Figure 4: **DNA binding specificity of the wild-type repressor.** The DNA binding specificity of the wild-type repressor was monitored on **a**) a 30-bp substrate that contains the consensus sequence (underlined), **b**) a 30-bp scrambled substrate that contains the same nucleotide content as the substrate in panel **a**) but lacks the consensus sequence, and **c**) a 13-bp substrate that contains only the consensus sequence. For each gel, the fluorescein-labeled DNA was mixed with 0, 0.02, 0.04, 0.08, 0.16, 0.31, 0.63, 1.25, 2.5, or 5μM protein. The mass of free DNA in the absence of protein (8.5 kDa or 19.0 kDa) is indicated for each gel. Non-specific DNA band migration is observed in the highest two protein concentrations and is not included in the analysis. The results show that the repressor only forms a complex with the substrate that contains both the consensus motif and flanking nucleotides around the consensus. Each gel is a representative of triplicate experiments that were performed using the wild-type protein. Source data for this figure are provided in Supplementary Figure 9.

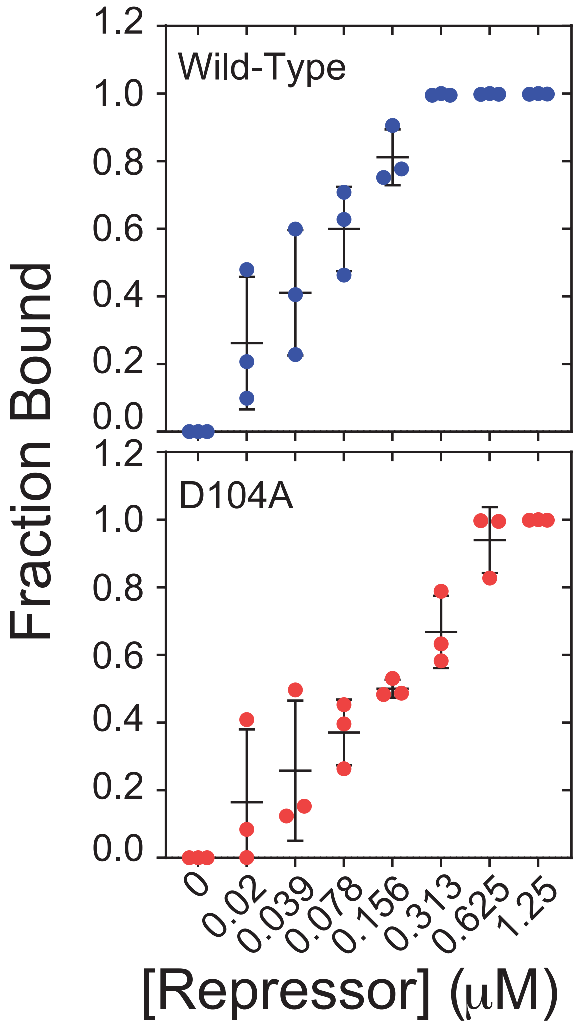

Supplementary Figure 5: **DNA binding data for the wild-type and D104A mutant repressors.** Plotted are all data points measured from three independent experiments for wild-type (blue) and D104A (red) repressors binding the optimal 30-bp DNA substrate, along with the mean and standard deviations at each protein concentration. These data were used to generate the plots shown in Figures 4c and 4d. Source data are provided in the Source Data file.

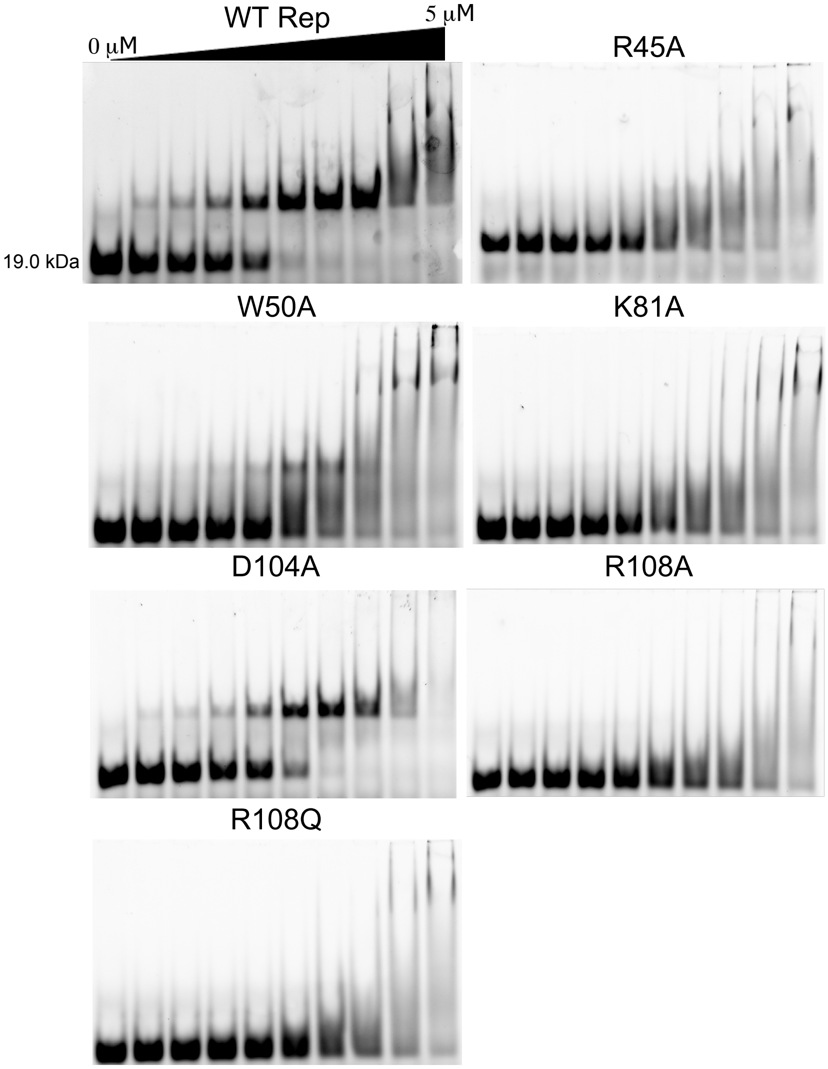

Supplementary Figure 6: **DNA binding properties of repressor point mutants.** Repressor point mutants that displayed a phenotype in the immunity assays were assessed for their ability to bind a 30-bp substrate that contains the consensus sequence. For each gel, the fluorescein-labeled DNA was mixed with 0, 0.02, 0.04, 0.08, 0.16, 0.31, 0.63, 1.25, 2.5, or 5 μM protein. The mass of free DNA in the absence of protein (19.0 kDa) used in all experiments is indicated for the wild-type gel. Non-specific DNA band migration is observed in the highest two protein concentrations. A titration of the wild-type repressor (WT Rep) shows a shift in band size indicative of protein:DNA complex formation. While the D104A mutant shows DNA binding activity that is similar to wild-type, all other mutants tested show a severe defect in forming a specific complex on DNA. We do see a small amount of specific complex formation with the W50A mutant, but the band smearing occurring between the unbound and bound DNA bands made this complex difficult to quantify. Each gel is a representative of triplicate experiments that were performed for the wild-type protein and each mutant. Source data for this figure are provided in Supplementary Figure 10.

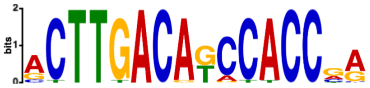

50816	TGGGCCTGAGA**CTTGACATCCACC**GAAGGGACGAGA
4258	CGTGTATGCAA**CTTGACATCCACC**GAGAAAGGCACA
38882	GCTGTCTCGAA**CTTGACATCCACC**AAGGAGGAAACA
50711	GTATCCGCTGA**CTTGACAGCCACC**GAAGGAGGCTAG
43088	AGTGAGGTCAA**CTTGACAGCCACC**GATACTCACCTT
42610	TCTGTGACCAA**CTTGACAGCCACC**GAGAGGAAGACA
40602	ACGCGCACCGA**CTTGACAGCCACC**GAGGAAAGGATC
36182	GACGGTGTGAA**CTTGACAGCCACC**AAGCTCCCGTAC
41225	CGTTAGTCCGA**CTTGACATCCACC**CAACAGAAAGTA
32370	CATGTCGACTA**CTTGACATCCACC**GGGACCGAGGTC
51144	TGGTTCTCAGG**CTTGACATCCACC**GAAGGGGTGAAC
18273	CATGTCGCTAA**CTTGACATCCACC**AGGAGGTAACCA
48722	CGTGCGCGCCG**CTTGACATCCACC**AAGAAAGGCAAG
51508	AAAACGGCCCG**CTTGACAGCCACC**GAAACATCCGGT
28764	TAGGAGGAGAA**CTTGACAGACACC**GAGCCGAGGAAG
14790	GCTGATCCAAA**CTTGACAGCCACC**CGGCTGTCTACC
12199	CTTGATCGCAG**CTTGACATCCACC**GGGAGGGGGCTC
45070	AAAGAGATACA**CTTGACAGGCACC**AACATGTCCCCG
50445	CGTGACGCCGA**CTTGACAGCCACC**ACGATTCTGCTG
48329	GCTGGTGACCC**CTTGACATCTACC**GAAGGAGAAGAC
6488	GTCGAGTCGAA**CTTGACATACACC**TGAAAGGAGGCG
189	AGGTGAGTATC**CTTGACAGCCACC**CGTCAGGAGTGG
45565	GAGTTGGACGA**CTTGACAGACTCC**GACTTCCAGTCC
51806	GGCGTAGTGCC**CTTTACAGCCACC**GATACGGGCGAA
51634	AGATTCTCCGG**TTTGACAGCCACC**CGGTTATCCGGT
51748	TTCAATTCGGG**CTTGACGTCCACC**CGGAGCTAGTGC

Supplementary Figure 7: **Operator/Stoperator sites in the TipsytheTRex genome.** The consensus sequence for TipsytheTRex, as identified using the MEME suite, is shown. Below the consensus are the 26 sites found in the TipsytheTRex genome, with the consensus motif of each sequence underlined and in bold. Nucleotides that represent deviations in the consensus are colored red. The sequences are ordered with respect to E-values calculated in MEME. The coordinate values identify the location of each sequence in the genome.

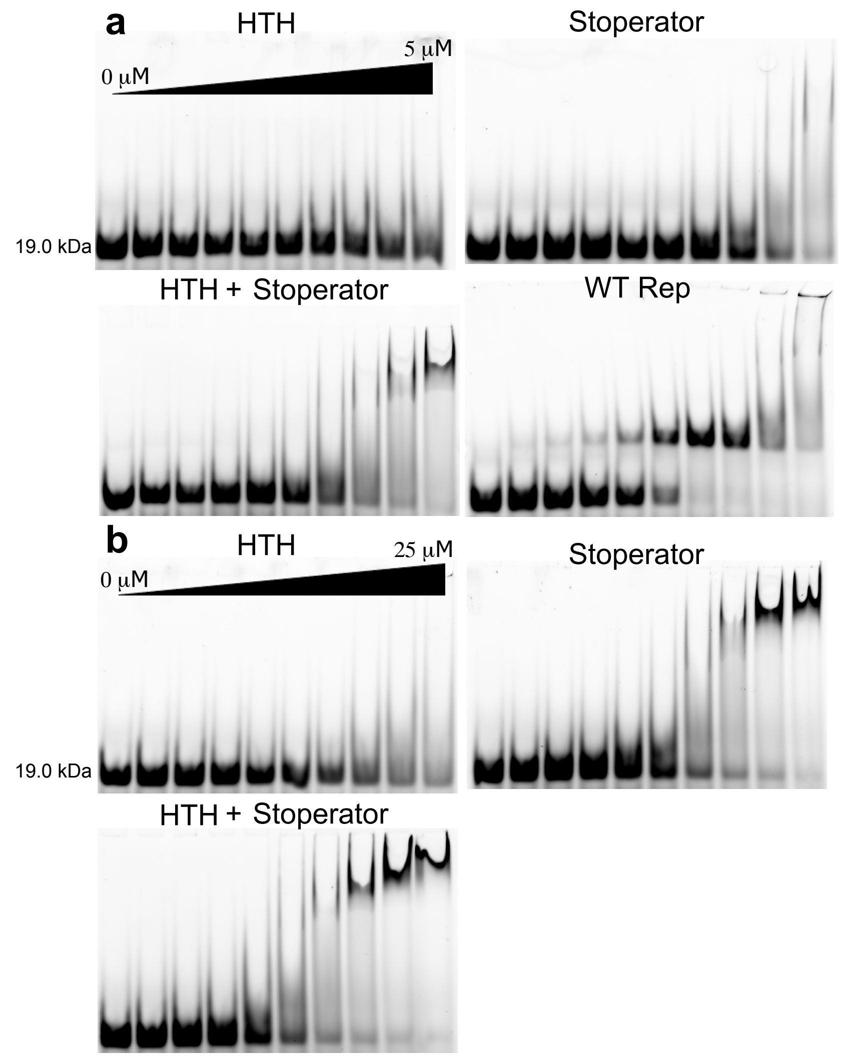

Supplementary Figure 8: **DNA binding properties of the HTH and Stoperator domains.** The HTH and Stoperator domains of the repressor were independently expressed and purified and their DNA binding properties assessed on a 30-bp substrate that contains the consensus sequence. **a**) The DNA binding properties of each indicated protein was monitored by mixing a fluorescein-labeled DNA with 0, 0.02, 0.04, 0.08, 0.16, 0.31, 0.63, 1.25, 2.5, or 5 μM protein. The mass of free DNA in the absence of protein (19.0 kDa) used in all experiments is indicated for the first gel in both panels **a**) and **b**). While the wild-type repressor (WT Rep) shows a shift in band size indicative of protein:DNA complex formation, the individual HTH and Stoperator domains fail to form a specific complex on DNA when assayed alone or in the presence of the other domain. The smearing present in the last two lanes of all gels represent non-specific complex formation present at high protein concentrations. **b**) The DNA binding properties of the HTH and Stoperator domains were monitored at a higher protein concentration range. For each gel, a fluorescein-labeled DNA was mixed with 0, 0.10, 0.20, 0.39, 0.79, 1.6, 3.1, 6.3, 12.5, or 25 μM protein. Despite the higher protein concentrations, a specific complex on DNA is not observed, and with the Stoperator domain we see a substantial amount of non-specific complex form at high protein concentrations. Each gel is a representative of triplicate experiments that were performed for each protein. Source data for this figure are provided in Supplementary Figure 11.

SAXS sample	D_{max} (Å)	R_g (Å) Guinier plot[a]	MW Seq. calculated (kDa)	MW[b] SAXS (kDa)	MW MALS (kDa)	Model fit χ^2
Repressor	~ 90	22.6± 0.7	23.6	29	17	2.1
Repressor + 13-bp DNA	~ 80	19.8± 0.7	31.7	13	15	1.2
Repressor + 24-bp DNA	~ 80	24.2± 0.4	38.5	31	27	1.6

Supplementary Table 1: Structural parameters from the SAXS and MALS data
[a]R_g and reported errors are derived from curve fits generated from Guinier plots
[b]SAXS-based Molecular weight was calculated based on Volume of Correlation (Vc)[2]

DNA Substrate	Oligo 1	Oligo 2
21-bp crystallization	TTTCGGTGGCTGTCAAGCGGG	CCCGCTTGACAGCCACCGAAA
24-bp SEC-SAXS-MALS	TGTTTCGGTGGCTGTCAAGCGGGC	GCCCGCTTGACAGCCACCGAAACA
13-bp SEC-SAXS-MALS	GGTGGCTGTCAAG	CTTGACAGCCACC
30-bp Optimal DNA Binding	ACGGCCCGCTTGACAGCCACCGAAACATCC	GGATGTTTCGGTGGCTGTCAAGCGGGCCGT-FAM
30-bp Scrambled DNA Binding	ACCCTGGAGAAATCCCCTAGCCCAGCCAGC	GCTGGCTGGGCTAGGGGATTTCTCCAGGGT-FAM
13-bp Optimal DNA Binding	CTTGACAGCCACC	GGTGGCTGTCAAG-FAM

Supplementary Table 2: Oligonucleotides used for crystallization, SEC-SAXS-MALS, and DNA binding studies. Each oligonucleotide sequence is written in the 5′-3′ direction. To generate each duplex substrate, oligo 1 was mixed with oligo 2 in an equimolar ratio in 10 mM Tris (pH 8.0), 50 mM NaCl, and 1 mM EDTA, heated to 95 C, and then allowed to cool to room temperature in a water bath. For the DNA binding studies, fluorescein (FAM) was added to the 3′-end of oligo 2.

Supplementary References

1. Robert, X. & Gouet, P. Deciphering key features in protein structures with the new ENDscript server. *Nucleic Acids Res* **42**, W320-4 (2014).
2. Rambo, R.P. & Tainer, J.A. Accurate assessment of mass, models and resolution by small-angle scattering. *Nature* **496**, 477-81 (2013).

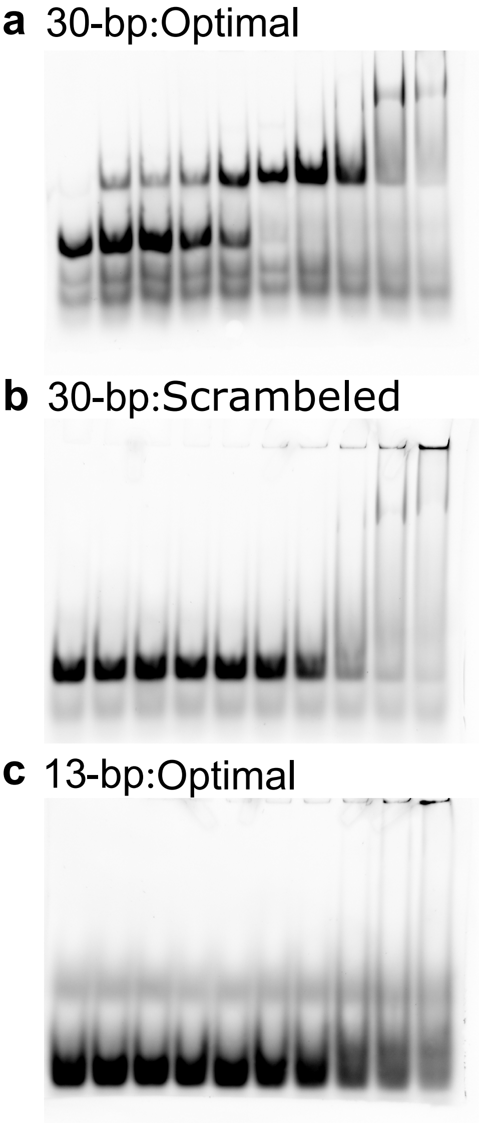

Supplementary Figure 9: Uncropped gel images used to generate Supplementary Figure 4. Shown are uncropped gels of the wild-type repressor binding the **a**) 30-bp optimal substrate, **b**) 30-bp scrambled substrate, and **c**) 13-bp optimal substrate.

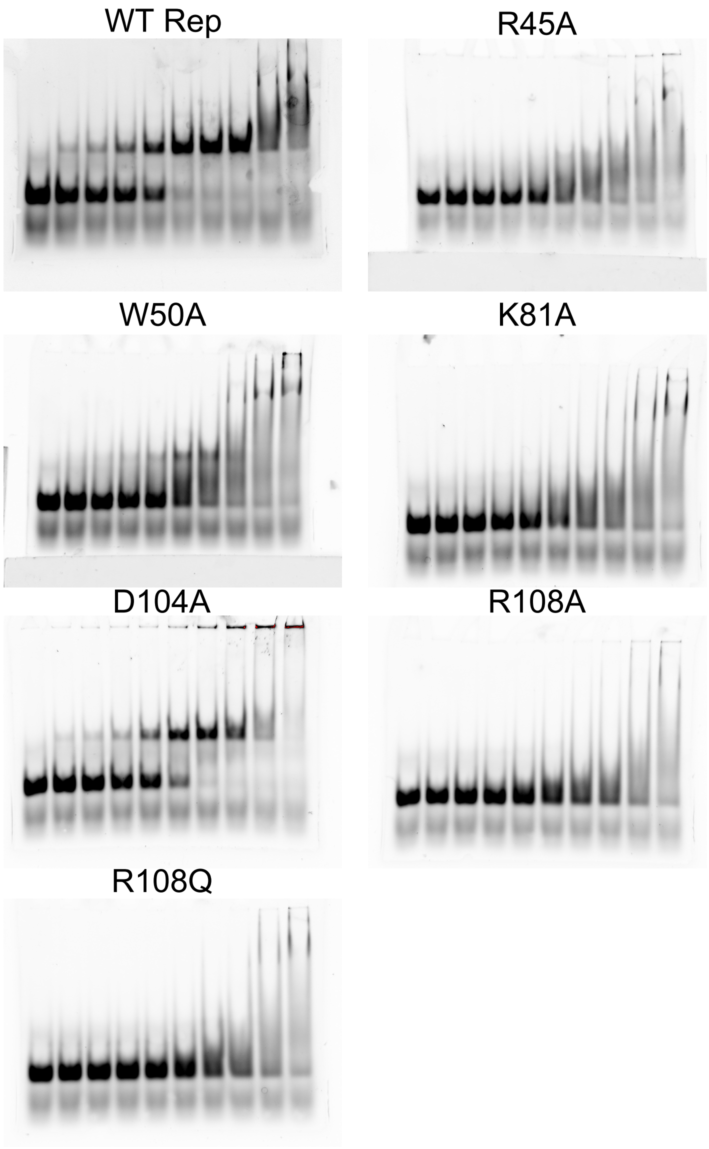

Supplementary Figure 10: Uncropped gel images used to generate Supplementary Figure 6. Shown are uncropped gels of the wild-type repressor (WT Rep) and the indicated point mutants binding the 30-bp optimal substrate.

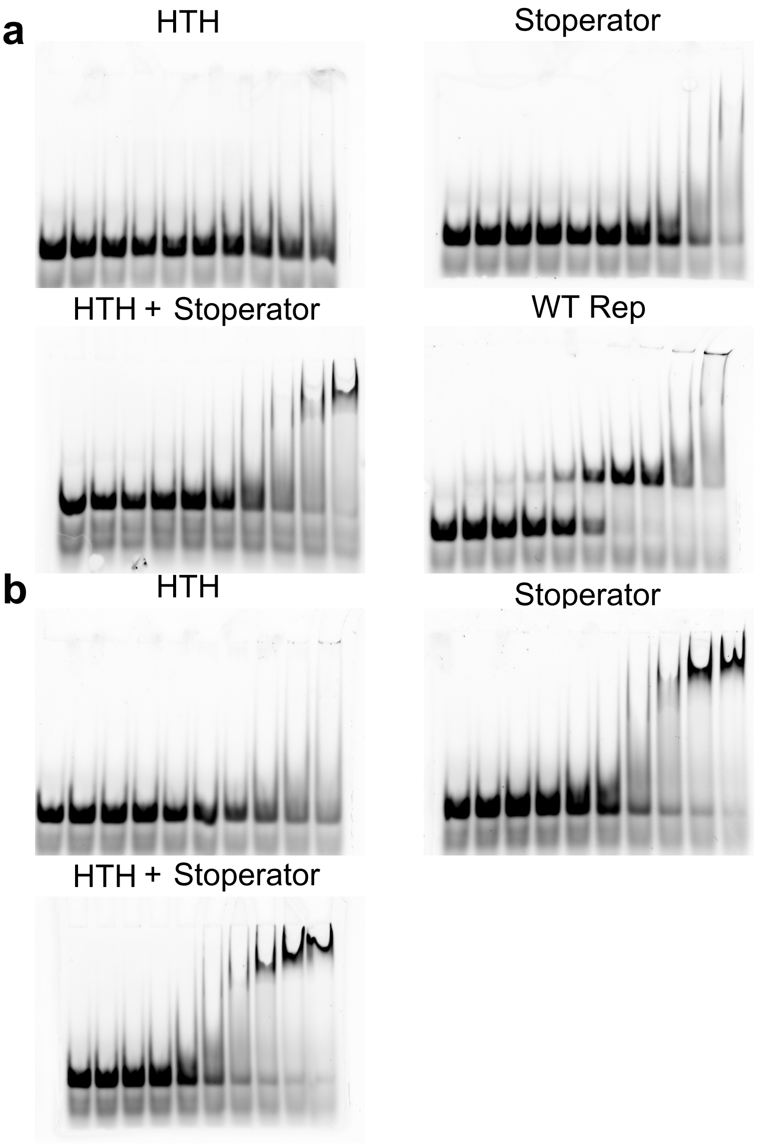

Supplementary Figure 11: Uncropped gel images used to generate Supplementary Figure 8. Shown are uncropped gels of the HTH domain, Stoperator domain, a mixture of the HTH and Stoperator domains, and the wild-type repressor (WT Rep) binding the 30-bp optimal substrate. **a**) The DNA binding properties of each indicated protein was monitored by mixing a fluorescein-labeled DNA with 0, 0.02, 0.04, 0.08, 0.16, 0.31, 0.63, 1.25, 2.5, or 5 μM protein. **b**) The DNA binding properties were monitored at a higher protein concentration range. For each gel, a fluorescein-labeled DNA was mixed with 0, 0.10, 0.20, 0.39, 0.79, 1.6, 3.1, 6.3, 12.5, or 25 μM of the indicated protein.
