# Supplementary material for: A monomeric mycobacteriophage immunity repressor utilizes two domains to recognize an asymmetric DNA sequence
Source: Nat Commun. 2022 Jul 14;13:4105. doi: 10.1038/s41467-022-31678-6 (PMC9283540; doi:10.1038/s41467-022-31678-6)
Supplement: Supplementary file 3 — Reporting Summary [file 41467_2022_31678_MOESM3_ESM.pdf]

nature portfolio

Corresponding author(s): Maria D. Gainey and Jamie R. Wallen

Last updated by author(s): Jun 8, 2022

Reporting Summary

Nature Portfolio wishes to improve the reproducibility of the work that we publish. This form provides structure for consistency and transparency in reporting. For further information on Nature Portfolio policies, see our Editorial Policies and the Editorial Policy Checklist.

Statistics

For all statistical analyses, confirm that the following items are present in the figure legend, table legend, main text, or Methods section.

n/a	Confirmed	
☐	☒	The exact sample size (*n*) for each experimental group/condition, given as a discrete number and unit of measurement
☐	☒	A statement on whether measurements were taken from distinct samples or whether the same sample was measured repeatedly
☒	☐	The statistical test(s) used AND whether they are one- or two-sided<br>*Only common tests should be described solely by name; describe more complex techniques in the Methods section.*
☒	☐	A description of all covariates tested
☒	☐	A description of any assumptions or corrections, such as tests of normality and adjustment for multiple comparisons
☐	☒	A full description of the statistical parameters including central tendency (e.g. means) or other basic estimates (e.g. regression coefficient) AND variation (e.g. standard deviation) or associated estimates of uncertainty (e.g. confidence intervals)
☒	☐	For null hypothesis testing, the test statistic (e.g. *F*, *t*, *r*) with confidence intervals, effect sizes, degrees of freedom and *P* value noted<br>*Give P values as exact values whenever suitable.*
☒	☐	For Bayesian analysis, information on the choice of priors and Markov chain Monte Carlo settings
☒	☐	For hierarchical and complex designs, identification of the appropriate level for tests and full reporting of outcomes
☒	☐	Estimates of effect sizes (e.g. Cohen's *d*, Pearson's *r*), indicating how they were calculated

Our web collection on statistics for biologists contains articles on many of the points above.

Software and code

Policy information about availability of computer code

Data collection	Wyatt Astra V7.1.48 software was used for UV, MALS, and RI data. For MD Data collection, the GROMACS V2019 software suite was used. RTA (real-time analysis) V1.18.54 software was used for whole-genome DNA sequencing data collection. Image Lab V5.2.1 software was used to collect gel images for the DNA binding studies.
Data analysis	Wyatt Astra V7.1.48 software was used for UV, MALS, and RI data. SAXS data were analyzed using Scatter V.e. For SAXS modeling, the programs MODELLER V9.25, BILBOMD V2.0, and FoxS (https://modbase.compbio.ucsf.edu/foxs/) were used. X-ray diffraction data were indexed, integrated, and scaled using XDS V20190315 and HKL3000 V721.3. For structure solution and refinement, Phenix V1.19.2-4158 and V1.20-4459 and CCP4i V1.0.2 were used. The structure was solved using the programs Crank2 (CCP4), AutoSol (Phenix), and Autobuild (Phenix). The structure was refined using Phenix, AlphaFold, Refmac (CCP4), and PDB-REDO, with model building performed in Coot V0.8.9.2. For MD data analysis, the GROMACS V2019, Bio3d V2.3-0, and Visual Molecular Dynamics (VMD) V1.9.4a51 software suites were used. Image Lab version 5.2.1 software was used for DNA binding analysis. The programs Newbler V2.9, Ace Util, and Consed V29 were used for genome assembly. Figures were prepared using the programs Inkscape 1.0, EsPript 3.0, Adobe Illustrator 2021 and 2022, Chimera X, and Pymol 2.3.0/2.4.1.

For manuscripts utilizing custom algorithms or software that are central to the research but not yet described in published literature, software must be made available to editors and reviewers. We strongly encourage code deposition in a community repository (e.g. GitHub). See the Nature Portfolio guidelines for submitting code & software for further information.

Data

Policy information about availability of data

All manuscripts must include a data availability statement. This statement should provide the following information, where applicable:

- Accession codes, unique identifiers, or web links for publicly available datasets
- A description of any restrictions on data availability
- For clinical datasets or third party data, please ensure that the statement adheres to our policy

Coordinates and structure factors for the native and selenomethionine crystal structures have been deposited in the PDB under accession codes 7R6R [http://doi.org/10.2210/pdb7R6R/pdb] and 7TZ1 [http://doi.org/10.2210/pdb7TZ1/pdb], respectively. SAXS data and atomistic models have been deposited in the SASBDB database as entries SASDMK3 (repressor only) [https://www.sasbdb.org/data/SASDMK3/], SASDML3 (Repressor:24-bp DNA) [https://www.sasbdb.org/data/SASDML3/], and SASDMM3 (Repressor:13-bp DNA) [https://www.sasbdb.org/data/SASDMM3/]. All MD data have been deposited in the Zenodo public repository [DOI: 10.5281/zenodo.6604542]. The TipsytheTRex genome sequence has been deposited in GenBank under accession code MF919536 [https://www.ncbi.nlm.nih.gov/nuccore/MF919536]. Whole-genome sequencing data have been deposited in the NCBI BioProject database under accession code PRJNA818041 [https://www.ncbi.nlm.nih.gov/bioproject/?term=PRJNA818041]. Source data are provided with this paper.

Field-specific reporting

Please select the one below that is the best fit for your research. If you are not sure, read the appropriate sections before making your selection.

☒ Life sciences ☐ Behavioural & social sciences ☐ Ecological, evolutionary & environmental sciences

For a reference copy of the document with all sections, see nature.com/documents/nr-reporting-summary-flat.pdf

Life sciences study design

All studies must disclose on these points even when the disclosure is negative.

Sample size	Sample sizes are indicated for each experiment and were chosen based on similar previous studies. Images for X-ray diffraction data were collected for maximum resolution and completeness (statistics provided in Table 1). Where statistical tests were applied (in vivo superinfection immunity assays, MD Data, and DNA binding studies), we selected n=3, which gave highly reproducible results . For DNA sequencing, we selected five independent plaques (n=5) to sequence and compare to wild-type.
Data exclusions	10% of the observed reflections in the X-ray diffraction experiments were excluded during crystal structure refinement in order to reduce model bias.
Replication	For the in vivo superinfection immunity assays, experiments were performed in triplicate, with highly reproducible results. All crystals were isomorphous, with nearly identical space groups. The protein was purified multiple times, with highly reproducible results. For the molecular dynamics simulations, experiments were performed in triplicate. Once the behaviors were established to be reproducible, steered molecular dynamics (SMD) methods were used to promote dissociation of the protein:DNA complex. Dissociation equilibrium constants and associated error were estimated by bootstrapping methods with n =200. For all DNA binding studies, experiments were performed in triplicate, with highly reproducible results.
Randomization	10% of reflections from each of the two diffraction data sets were randomly allocated by Phenix for Rfree calculations. All other experiments in this study did not include randomized experiments, and the result were not randomized. For X-ray crystallography, covariates were controlled by using Rwork/Rfree values. For SEC-SAXS, covariates were controlled by the use of SEC prior to SAXS data collection, which ensured the analysis of a monodispersed sample. For in vivo superinfection immunity and DNA binding assays, covariates were controlled by performing all experiments in the presence of controls. Additionally, all mutants generated were confirmed to express and purify similar to the wild-type protein.
Blinding	Blinding is not relevant/possible for our studies, as all experimental conditions performed were necessary for data analysis.

Reporting for specific materials, systems and methods

We require information from authors about some types of materials, experimental systems and methods used in many studies. Here, indicate whether each material, system or method listed is relevant to your study. If you are not sure if a list item applies to your research, read the appropriate section before selecting a response.

Materials & experimental systems

n/a	Involved in the study
☒	☐ Antibodies
☒	☐ Eukaryotic cell lines
☒	☐ Palaeontology and archaeology
☒	☐ Animals and other organisms
☒	☐ Human research participants
☒	☐ Clinical data
☒	☐ Dual use research of concern

Methods

n/a	Involved in the study
☒	☐ ChIP-seq
☒	☐ Flow cytometry
☒	☐ MRI-based neuroimaging
