# Supplementary material for: A monomeric mycobacteriophage immunity repressor utilizes two domains to recognize an asymmetric DNA sequence
Source: Nat Commun. 2022 Jul 14;13:4105. doi: 10.1038/s41467-022-31678-6 (PMC9283540; doi:10.1038/s41467-022-31678-6)
Supplement: Supplementary file 4 — Description of Additional Supplementary Files [file 41467_2022_31678_MOESM4_ESM.pdf]

Description of Additinoal Supplementray files

File name: Supplementary Video 1
Description: Essential dynamics corresponding to Principle Component 1 for a Molecular Dynamics simulation for apo repressor protein. Protein is represented in surface view.

File name: Supplementary Video 2
Description: Essential dynamics corresponding to Principle Component 2 for a Molecular Dynamics simulation for apo repressor protein. Protein is represented in surface view.

File name: Supplementary Video 3
Description: Essential dynamics corresponding to Principle Component 1 for a Molecular Dynamics simulation for repressor protein bound to DNA. Protein is represented in surface view.

File name: Supplementary Video 4
Description: Essential dynamics corresponding to Principle Component 2 for a Molecular Dynamics simulation for repressor protein bound to DNA. Protein is represented in surface view.
